# Characteristics of *PTEN* Mutation in Thyroid Tumours: A Retrospective Chart Review

**DOI:** 10.3390/cancers15051575

**Published:** 2023-03-03

**Authors:** Saruchi Bandargal, Mohannad Rajab, Véronique-Isabelle Forest, Marc Philippe Pusztaszeri, Michael P. Hier, Sabrina Daniela da Silva, Richard J. Payne

**Affiliations:** 1 Faculty of Medicine, McGill University, Montreal, QC H3G 2M1, Canada; 2 Department of Otolaryngology—Head and Neck Surgery, King Faisal Specialist Hospital & Research Center, Al Madinah Al Munawwarah 42523, Saudi Arabia; 3 Department of Otolaryngology—Head and Neck Surgery, McGill University, Jewish General Hospital, Montreal, QC H3T 1E2, Canada; 4 Department of Pathology, McGill University, Jewish General Hospital, Montreal, QC H3T 1E2, Canada; 5 Department of Otolaryngology—Head and Neck Surgery, McGill University, Royal Victoria Hospital, Montreal, QC H4A 3J1, Canada

**Keywords:** *PTEN* mutation, thyroid cancer, ThyGeNEXT, ThyroSeq v3, differentiated thyroid cancer

## Abstract

**Simple Summary:**

*PTEN* mutation is an extremely rare mutation in thyroid nodules with no clear prognostic indicators. In this multicenter study of 16 *PTEN*-mutated thyroid nodules, we found that 37.5% of the nodules were malignant. Aggressive features were present in 33.33% of the malignant tumours. We hypothesize that with time, *PTEN*-mutated thyroid nodules can acquire high allele frequencies (AFs) and widespread copy number alterations (CNAs), which might be aggravating the effects of *PTEN* mutations.

**Abstract:**

While some studies suggest that *PTEN* mutations correlate with a low-risk phenotype in pediatric thyroid nodules, the relationship between the mutation and malignancy in the adult populations is abstruse. This study investigated whether *PTEN* mutations result in thyroid malignancy, and whether these malignancies are aggressive. This multicenter study involved 316 patients who underwent preoperative molecular testing, and subsequent lobectomy or total thyroidectomy at two quaternary care hospitals. A four-year retrospective review was performed on the 16 charts of patients that opted for surgery following a positive *PTEN* mutation on molecular testing results from January 2018 to December 2021. Of the total 16 patients, 37.5% (*n* = 6) had malignant tumours, 18.75% (*n* = 3) had non-invasive follicular thyroid neoplasms with papillary-like nuclear features (NIFTPs), and 43.75% (*n* = 7) had benign disease. Aggressive features were detected in 33.33% of the malignant tumours. Malignant tumours were found to have a statistically significant higher allele frequency (AF). The aggressive nodules were all poorly differentiated thyroid carcinomas (PDTCs) with copy number alterations (CNAs) and the highest AFs.

## 1. Introduction

The rapid increase in thyroid cancer incidence, especially since 1990, despite the startling steadiness in mortality rates, suggests that the rate of aggressive thyroid cancer has not increased [1]. The largest escalation is prominent in middle-aged women, while incidence rates in men have varied significantly less. There exist multiple subtypes of thyroid malignancies, among which differentiated thyroid cancer (DTC) is the most frequent subtype, which the WHO subdivides into papillary, follicular, and oncocytic thyroid carcinoma [2]. The development of thyroid tumours involves the procurement of different genetic alterations that are associated with distinct gene expressions and signaling pathways that eventually lead to the development of idiosyncratic histophenotypes with different behaviors and prognoses [3]. Phosphatase and tensin homolog (PTEN) gene mutation is one of the genes found to be associated with benign and malignant thyroid tumours [4].

PTEN is a negative regulator of one of the most crucial cancer pathways: the phosphatidylinositol 3-kinase (PI3K)-AKT signaling pathway, which promotes cell proliferation and survival [5]. The loss of PTEN’s function permits certain cells to divide uncontrollably, contributing to tumourigenesis [6,7,8]. Germline *PTEN* mutations, inherited with an autosomal dominant mechanism, can lead to PTEN hamartoma tumour syndrome (PHTS), which is characterized by various benign and malignant tumours of the thyroid, breast, endometrium, and other organs [9]. PHTS comprises a spectrum of disorders with shared and distinct clinical features, including Cowden syndrome, Bannayan-Riley-Ruvalcaba syndrome, adult Lhermitte-Duclos disease, and autism spectrum disorders associated with macrocephaly [9,10,11].

Patients with PHTS are associated with an increased lifetime risk of thyroid cancer, which can range between 3% and 14% [12]. Children with phosphatase and tensin homolog (PTEN) gene mutations have a heightened risk of developing DTC [13]. The literature reports an incidence of 4–12% of DTC in children affected by *PTEN* mutations, mainly follicular, and up to 50% of benign nodular thyroid disease [14,15,16]. Because of the increased risk of thyroid malignancy in these patients compared to the general population, active surveillance and close follow-up are recommended [7,17].

However, most *PTEN* mutations detected via molecular testing in thyroid nodules are somatic and are associated with a limited ability to identify patients with PHTS [18]. In Quaytman et al.’s study, only 4 (8%) out of 49 *PTEN*-mutated thyroid nodules had germline mutations [19]. Whereas PHTS has been extensively researched, the characteristics and prognostic indicators of somatic *PTEN*-mutated thyroid nodules and cancers are yet to be elucidated. In previously published studies, most somatic *PTEN* mutations were associated with follicular patterned thyroid tumours. When such tumours are cancerous, they often result in low-risk malignancies; however, less frequently, *PTEN* mutations have been detected in poorly differentiated thyroid carcinomas (PDTCs) and anaplastic thyroid carcinomas (ATCs) [20,21,22,23,24]. Nonetheless, the factors that underpin these rare malignancies are nebulous.

Currently, some aggressive features of thyroid cancers can only be accurately identified postoperatively on histologic specimens. New methods to identify thyroid tumours with a higher risk of having such features could lead to improvements in the stratification of patient risk, better clinical management, and a decline in futile over-treatment. Molecular testing of thyroid cells is a tool that has shown promise in predicting malignancy and cancer aggressiveness preoperatively [3,25]. Additionally, it has emerged as an efficacious, ancillary tool for evaluating cytologically indeterminate thyroid tumours and guiding clinical management in cases where otherwise, management decisions are obscure [26]. However, to effectively translate a molecular testing result into more meaningful clinical practice, further information is required on the outcomes of thyroid tumours with various genetic alterations to establish coherent prognostic indicators. Our study described the clinicopathological features of *PTEN*-mutated thyroid tumours and investigated potential factors that can lead to malignancy and aggressive cancer.

## 2. Materials and Methods

### 2.1. Study Design

Our study is a multicenter retrospective chart review involving 316 lobectomy and total thyroidectomy patients that previously underwent molecular testing at two quaternary-level hospitals (Jewish General Hospital and Royal Victoria Hospital) in Montreal, Canada. Data on patient demographics, preoperative ultrasound-guided fine needle aspiration biopsy (USFNA), molecular diagnostic testing, and postoperative histopathology was compiled. Ethics approval was obtained by the Medical-Bioethics Research Ethics Committee (REC) of the Integrated Health and Social Services Network for West-Central Montreal (#MP-05-2022-3178).

### 2.2. Patient Population

The charts of adult patients (≥18 years of age) who underwent preoperative molecular diagnostic testing using ThyGeNEXT or ThyroSeq v3 followed by surgery between January 2018 and December 2021 were reviewed. A general written consent form employed at our institution for all surgery, anesthesia, diagnostic, or therapeutic procedures was used for acquiring consent for molecular testing and subsequently, surgery. Only patients who had tested positive for a *PTEN* mutation and underwent surgery were included in our study. Fourteen patients had a lobectomy, while two had a completion thyroidectomy. Additionally, all included patients had a sentinel lymph node biopsy and a limited central neck dissection.

All patients with thyroid nodules were evaluated using ultrasound imaging. Thyroid imaging, reporting, and data system (TI-RADS) criteria were used to classify the nodules and to identify nodules that were eligible for USFNA [27]. Then, patients were eligible for molecular testing if they had a Bethesda III or Bethesda IV nodule. Alternatively, molecular testing was warranted for patients with a Bethesda V or Bethesda VI nodule if, after consideration of clinical and sonographic features, the test result was expected to alter surgical decision-making, accordant with the 2015 American Thyroid Association Management Guidelines [28,29].

### 2.3. Tumour Analysis

Two thyroid nodule samples were collected for each patient using USFNA. One sample was transported to a commercial laboratory at the University of Pittsburgh Medical Center (UPMC) or Interpace Diagnostics for ThyroSeq v3 or ThyGeNEXT, respectively. These samples were analyzed for molecular alterations at the aforementioned laboratories. The second sample was sent to the pathology department at the affiliated hospitals for analysis, and reported according to the Bethesda system for reporting thyroid cytology [30].

Board-certified head and neck fellowship-trained pathologists reviewed the surgical resection specimens for aggressive features. The tumour nodules were examined completely and diagnosed according to the 2017 World Health Organization (WHO) classification of endocrine tumours [31]. Aggressive features were defined by the presence of one or more of the following: macroscopic extrathyroidal extension (ETE), lymph node metastasis (LNM), poorly differentiated thyroid carcinoma (PDTC), and high-risk histological features (tall cell, columnar cell, hobnail/micropapillary, and diffuse sclerosing).

Three study groups were established based on postoperative pathology diagnosis: patients with a benign nodule, those with a non-invasive follicular thyroid neoplasm with papillary-like nuclear features (NIFTP), and those with a malignant nodule.

### 2.4. Statistical Analysis

Descriptive statistics were conducted. Statistical analyses of associations between variables were performed using the two-sided Fisher’s exact test (with a significance set for *p* < 0.05), and for continuous variables, the non-parametric Mann–Whitney U test was utilized. All analyses were performed using the statistical software package STATA-13 (STATA Corporation, College Station, TX, USA).

## 3. Results

### 3.1. Baseline Characteristics

Of the total 316 patients screened, 16 harboured a *PTEN* mutation and were included in our study, resulting in a mutation frequency of 5%. Baseline information was calculated for each tumour: age, sex, the longest axis measurement of the tumour as per the postoperative pathology report, and the USFNA results using the Bethesda classification. The clinical and pathologic features of all 16 patients with full diagnostic characteristics are shown in Table 1.

### 3.2. Cytology

Most of the surgically resected *PTEN*-mutated thyroid tumours were diagnosed as Bethesda III, with 8 of 16 (50%) thyroid nodules belonging to the atypia of undetermined significance or follicular lesion of undetermined significance (AUS/FLUS) category. Five tumours (31.2%) were diagnosed as Bethesda IV or follicular neoplasm or suspicious for a follicular neoplasm (FN/SFN), two tumours (12.5 %) were Bethesda V or suspicious for malignancy, and one tumour (6.3%) was Bethesda VI or malignant.

### 3.3. Tumour Characteristics

**Figure 1:**
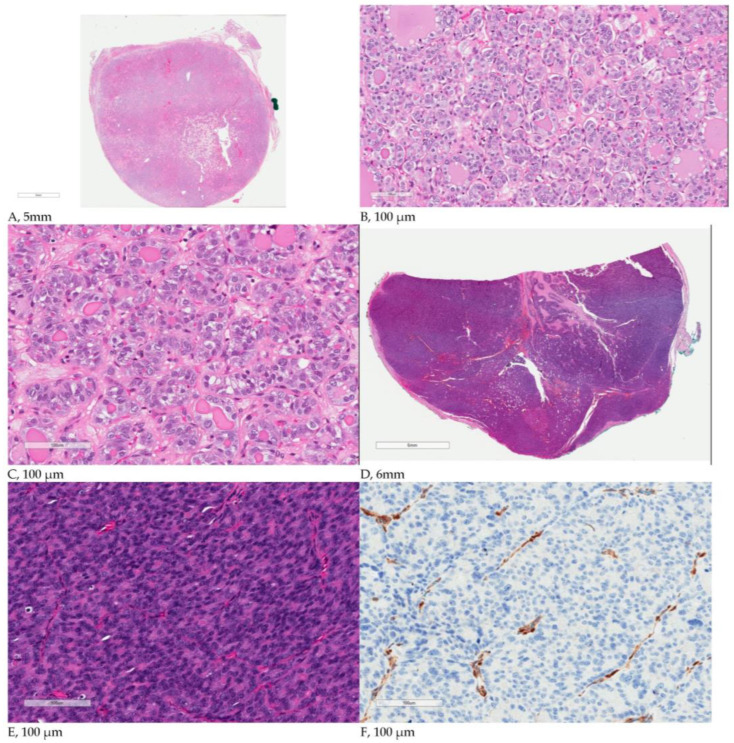
(**A**–**C**) A case of follicular variant of papillary thyroid carcinoma (PTC) ((**A**), low magnification) showing follicular growth pattern ((**B**), high magnification) with a minor solid/trabecular component ((**C**), high magnification). Note the well-developed nuclear features of PTC, including enlarged and ovoid nuclei, chromatin clearing, and nuclear membrane irregularities with grooves. (**D**–**F**) A case of poorly differentiated thyroid carcinoma showing a solid growth pattern ((**D**), low magnification) with tumour cells that have a high nuclear/cytoplasmic ratio and smaller, darker nuclei without the nuclear features of PTC ((**E**), high magnification). Areas with mitotic activity and focal necrosis (not shown) were present in this case. Immunohistochemistry with PTEN ((**F**), high magnification) demonstrates the loss of PTEN expression in the tumour cells, with a positive control in the endothelial cells, in keeping with the *PTEN* mutation. There was limited vascular invasion. The Ki-67 was 5–10%.

Of the 16 nodules with a *PTEN* mutation, 7 were benign, 3 were NIFTPs, and 6 were malignant. Two malignant tumours demonstrated aggressive features. Both aggressive tumours had areas of PDTC. As expected, these tumours demonstrated a loss of PTEN expression in the tumour cells according to immunohistochemistry (Figure 1).

**Figure 2:**
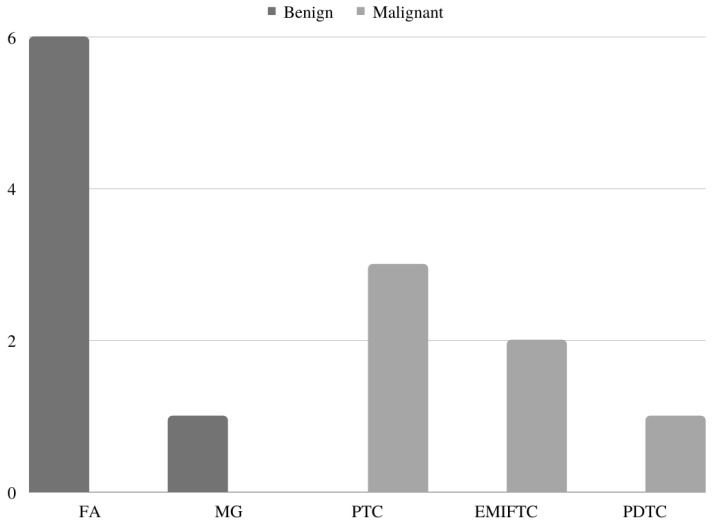
The frequency of histopathologic diagnoses by study group. FA: follicular adenoma; MG: multinodular goiter; PTC: papillary thyroid carcinoma; EMIFC: encapsulated minimally invasive follicular thyroid carcinoma; PDTC: poorly differentiated thyroid carcinoma.

The mean tumour size for the benign group was 1.5 cm, 1.8 cm for the NIFTP group, and 2.9 cm for the malignant group. The diagnoses for the groups are as follows, and are summarized in Figure 2: six follicular adenomas (FAs) and one multinodular goiter (MG) for the benign group; for the malignant group: three papillary thyroid carcinomas (PTCs), two encapsulated minimally invasive follicular thyroid carcinomas (EMIFTCs) and one PDTC.

The malignant group demonstrated a significantly higher mean allele frequency (AF) (41.8%) in comparison to the benign group with an AF of 18.3%. The NIFTP group harboured an AF of 36.5%. Furthermore, the two highest AFs belonged to the two aggressive tumours (70% and 91%). When excluding the two aggressive tumours’ AFs from the malignant group, the mean AF declined to 22.5%. Four tumours and one NIFTP had copy number alterations (CNAs). Both the aggressive tumours had CNAs. No tumour possessed co-existing genetic mutations.

## 4. Discussion

In the past decade, molecular testing of thyroid nodules has been rapidly evolving. It is becoming a valid tool in the routine workup of thyroid nodules [3]. It may serve as a valuable tool for triaging indeterminate nodules into those requiring surveillance from those requiring surgery [26]. Furthermore, it can be useful in optimizing decision-making for Bethesda V and VI thyroid nodules [28]. Our study’s goal was to determine whether *PTEN* mutations result in thyroid malignancy and aggressive phenotypes. While a clear genotype–phenotype correlation has not been established with regard to *PTEN* mutation aggressivity, there is some evidence that mutations leading to stable but inactive proteins produce a more adverse phenotype than mutations leading to proteins with partially retained function [7]. Furthermore, PTEN protein loss is more frequent in tumourigenesis than in *PTEN* genetic alterations [32].

To our knowledge, our study is the second study in the literature to discuss the characteristics of *PTEN* mutation in thyroid nodules that were identified with preoperative molecular testing. In Quaytman et al.’s study, they included 48 patients with *PTEN*-mutated thyroid nodules which were detected using ThyroSeq v3. Of their 48 patients, 20 were treated with surgery (either lobectomy or total thyroidectomy) and 28 underwent active surveillance. Almost all the 20 patients who underwent surgery had benign tumours (95%) (14 FAs, 4 oncocytic adenomas and 1 oncocytic hyperplastic nodule), and only one patient had a malignant tumour (5%), which was a low risk encapsulated follicular variant of papillary thyroid carcinoma (FVPTC) with no invasive features. They concluded that isolated somatic *PTEN*-mutated thyroid tumours are predominantly benign and are unlikely to grow at a high rate [19]. However, our study showed that seven patients (43.8%) had benign tumours, three patients (18.7%) had NIFTPs, and six patients (37.5%) had malignant tumours with two of them having aggressive features (33.3%). Although Quaytman et al. suggested that the threshold of surgery can be elevated for thyroid nodules with isolated *PTEN* mutations because of the low risk of malignancy in their study, our findings demonstrated that more than half of all the *PTEN*-mutated thyroid nodules in our study were either NIFTPs or malignant tumours that require surgical intervention.

*PTEN* mutation is a very rare mutation detected in thyroid cancer as demonstrated by our study’s mutation frequency of 5%, which is congruous to the frequencies reported in the literature [12,32]. Multiple studies evaluated the molecular profiles of different types of thyroid cancers, including PTC, FTC, NIFTP, and PDTC [15,19,21,22,23,33]. Our findings regarding the final tumour types of *PTEN*-mutated thyroid nodules are comparable with the literature. Most *PTEN*-mutated thyroid tumours are follicular patterned, and when malignant, they are predominantly low-risk cancers. Seldom, they can be aggressive, with poorly differentiated components. The majority of the tumours were FAs, which is in accord with Quaytman et al.’s results in their study comprising germline and somatic *PTEN*-mutated thyroid nodules [19]. Furthermore, we discovered that all benign nodules were CNA-negative. We found that *PTEN* mutations are associated with malignancy 37.5% of the time. *PTEN* mutations mostly lead to benign tumours, NIFTPs, and DTCs that lack aggressive features.

All the aggressive tumours in our study contained poorly differentiated components in tandem with CNAs and high AFs. CNAs can play a role in promoting tumour progression by altering the gene expression levels of genes located in the affected genomic regions. However, their presence does not always translate proportionally into altered expression levels due to transcriptional adaptive mechanisms [12,34]. Both PDTCs had the highest AFs (70% and 91%). AF is the number of mutant molecules over the total number of wild-type molecules at a specific location in the genome. The literature hypothesizes, in accordance with preliminary data, that AF in part would act as a surrogate of tumour burden, and therefore, the highest AF would negatively correlate with prognosis and overall survival [35]. AFs typically go up to 50%, indicating that double that percentage of cells can have the mutation. However, the two aggressive tumours had AFs surpassing 50%, which could suggest an amplification of the mutant allele or potential undetected germline alterations. In our study, the three cases with the highest AFs (45%, 70% and 91%) showed a pattern of continuity that started with solid architecture in the tumour with 45% AF and ended with the presence of the full diagnostic criteria of PDTC in the tumour with 91% AF (Figure 2). The tumour with 70% AF laid in between with a 5–10% component of PDTC. We hypothesize that with time, *PTEN*-mutated thyroid nodules can acquire high AFs and widespread CNAs, which might be aggravating the effects *PTEN* mutations, leading to more aggressive tumours.

The guidelines for the management of thyroid nodules have been set out by multiple studies. In a study conducted on pediatric patients with PHTS, the authors recommended surveillance for DTC from the age of 10 years onwards. They proposed surveillance to include yearly neck palpations and triennial thyroid ultrasounds [36]. The literature has extensively proposed recommendations for patients with PHTS, which is characterized by multiple hamartomas or benign tumour-like malformations throughout the body [12,16,37,38]. However, peer-reviewed recommendations targeted specifically for *PTEN*-mutated thyroid cancer are scarce. The 2015 ATA management guidelines for adult patients with thyroid nodules and differentiated thyroid cancer also reference *PTEN* in concurrence with PHTS, and do not utilize molecular markers as inclusion or exclusion criteria for active surveillance [28]. The guidelines suggest hemithyroidectomy or total thyroidectomy for 1–4 cm well-differentiated thyroid cancers with no evidence of preoperative high-risk disease. Furthermore, they suggest active surveillance as an alternative to immediate surgical excision in patients with low-risk papillary thyroid microcarcinomas (PTMCs) and in some cases of indeterminate thyroid nodules [28]. Some studies showed that even with strict adherence to the guidelines, the completion rate in 1–4 cm well-differentiated thyroid cancers with no evidence of preoperative high-risk features was still high (35–65%) [39,40,41,42]. Other studies showed that PTMCs can be associated with a 18.7% risk of aggressive features [43]. Multiple studies have demonstrated the potential of molecular testing to help in decision-making for thyroid nodules in certain situations, including indeterminate thyroid nodules and deciding on the extent of the surgery in Bethesda V and Bethesda VI nodules [3]. The two aggressive cases in our study were found to be Bethesda IV and Bethesda V on USFNA results with no preoperative evidence for aggressive disease. We founsd that malignant tumours had a significantly higher AF. Due to the small sample size, we could not definitively identify any other factors that are related to the risk of malignancy and aggressiveness in *PTEN*-mutated thyroid nodules which can help in optimizing the management of these nodules. However, we hypothesize that CNAs and higher AFs might play a role in elevating the aggressivity of *PTEN*-mutated thyroid nodules. Further studies with a larger sample size are requisite to uncover such determinants.

Our study harbours some limitations, including the inherent limitations of a retrospective study. Prospective studies are requisite to optimize and validate thresholds for clinical application. An unequivocal study limitation is the small sample size; however, *PTEN* mutations in thyroid cancer are extremely rare as suggested by the literature, and our mutation frequency is in accordance with those of previously published studies. Our study is one of the few to assess *PTEN*’s mutation frequency in a patient population independent of PHTS. This study was denuded of ascertaining recurrence rates as we did not assess follow-up information. Additionally, our study is susceptible to selection bias since molecular testing costs were paid for by the patient. Therefore, not all patients that potentially had a *PTEN* mutation underwent molecular testing, underrepresenting the number of *PTEN*-mutated thyroid nodules in this study. As our quaternary care hospitals are located in the urban city of Montreal, Canada, a geographic selection bias, as well as a referral selection bias leaning towards aggressive malignancies were maybe introduced. Lastly, it is worth noting that two patients underwent ThyGeNEXT, which does not report an AF or the presence of CNAs.

## 5. Conclusions

Most of the thyroid nodules with *PTEN* mutations were either benign, NIFTPs or low-risk malignant tumours. Hence, *PTEN*-mutated thyroid nodules preferentially present a non-aggressive phenotype. However, 33.33% of the malignant tumours were PDTCs that were associated with a high AF and CNAs. Further studies with a larger sample size and a longer follow-up time are required to identify other factors that might be related to the risk of malignancy and aggressiveness of *PTEN*-mutated thyroid tumours.

## Figures and Tables

**Table 1:** Clinicopathological features of *PTEN*-mutated thyroid tumours by tumour type.

Case	Sex	Age (Yr)	Cytologic Diagnosis (Bethesda Score)	Histopathologic Diagnosis	Nodule Size (cm)	AF (%)	Presence of CNAs?	Aggressive Feature
1	F	77	IV	FA	1.5	17	No	-
2	F	68	IV	FA	1.8	22	No	-
3	F	45	III	FA	0.8	14	No	-
4	F	69	III	FA	1	17	No	-
5	F	39	III	MG	-	25	No	-
6 *	F	28	III	FA	2.3	-	-	-
7	F	43	III	FA	1.6	15	No	
8 *	F	45	III	NIFTP	1.5	-	-	-
9	F	54	III	NIFTP	2	37	Yes	-
10	M	62	VI	NIFTP	1.8	36	No	-
11	F	69	V	SVPTC	3.6	45	Yes	-
12	F	29	IV	FVPTC	4.3	70	Yes	5–10% component of PDTC
13	F	58	IV	EMIFTC	2.7	17	No	-
14	M	55	V	PDTC	3	91	Yes	PDTC with lymph- vascular invasion
15	F	75	III	FVPTC	2	17	Yes	-
16	F	73	IV	EMIFTC	2	11	No	-

Cases 1–7: Benign nodules; Cases 8–10: NIFTPs; Cases 11–16: Malignant nodules. * Patients underwent ThyGeNEXT molecular testing. AF: allele frequency; CNAs: copy number alterations; FA: follicular adenoma; MG: multinodular goiter; NIFTP: non-invasive follicular thyroid neoplasm with papillary-like nuclear features; SVPTC: solid variant of papillary thyroid carcinoma; FVPTC: follicular variant of papillary thyroid carcinoma; EMIFTC: encapsulated minimally invasive follicular thyroid carcinoma; PDTC: poorly differentiated thyroid carcinoma.

## Data Availability

The data presented in this study are available upon request from the corresponding author. The data are not publicly available due to the ethics approval agreement.
